# Trends in industrialization and commercialization of IgY technology

**DOI:** 10.3389/fimmu.2022.991931

**Published:** 2022-10-20

**Authors:** Saeed Yakhkeshi, Rao Wu, Brindha Chelliappan, Xiaoying Zhang

**Affiliations:** ^1^ College of Biological Science and Engineering, Shaanxi University of Technology, Hanzhong, Shaanxi, China; ^2^ Department of Stem Cells and Developmental Biology, Cell Science Research Center, Royan Institute for Stem Cell Biology and Technology, Academic Center for Education, Culture, and Research (ACECR), Tehran, Iran; ^3^ Department of Biomedical Sciences, Ontario Veterinary College, University of Guelph, Guelph, ON, Canada; ^4^ Centre of Molecular & Environmental Biology, Department of Biology, University of Minho, Braga, Portugal

**Keywords:** egg yolk antibody (IgY), IgY patent, IgY product, IgY company, industrialization, commercialization

## Abstract

IgY technology refers to the strategic production process involved in generating avian immunoglobulin (IgY) against target antigens in a much more cost-effective manner with broad applications in the fields of diagnostics, prophylaxis, and therapeutics for both human and veterinary medicine. Over the past decade, promising progress in this research area has been evident from the steep increase in the number of registered manufacturing companies involved in the production of IgY products, the number of patents, and the notable number of clinical trials underway. Hence, it is crucial to conduct a prospective analysis of the commercialization and marketing potential of IgY-based commercial products for large-scale applications. This review revealed that the number of IgY patent applications increased steeply after 2010, with the highest of 77 patents filed in 2021. In addition, 73 industries are reportedly involved in marketing IgY products, out of which 27 were promoting biotherapeutics for human and veterinary medicine and 46 were in the diagnostic field. IgY antibodies are being used as primary and secondary antibodies, with approximately 3729 and 846 products, respectively. Biotherapeutic product consumption has notably increased as a food supplement and as a topical application in human and veterinary medicine, which are under different clinical phases of development to reach the market with around 80 and 56 products, respectively. In contrast, the number of IgY products as parenteral administrations and licensed drugs is not well developed given the lack of technical standards established for IgY registration and industrialization, as well as the restriction of the nature of polyclonal antibodies. However, recent ongoing research on functional IgY fragments indicates a promising area for IgY applications in the near future. Therefore, retrospective analysis with speculations is mandatory for IgY technology maturation toward industrialization and commercialization.

## Introduction

**Figure 1:**
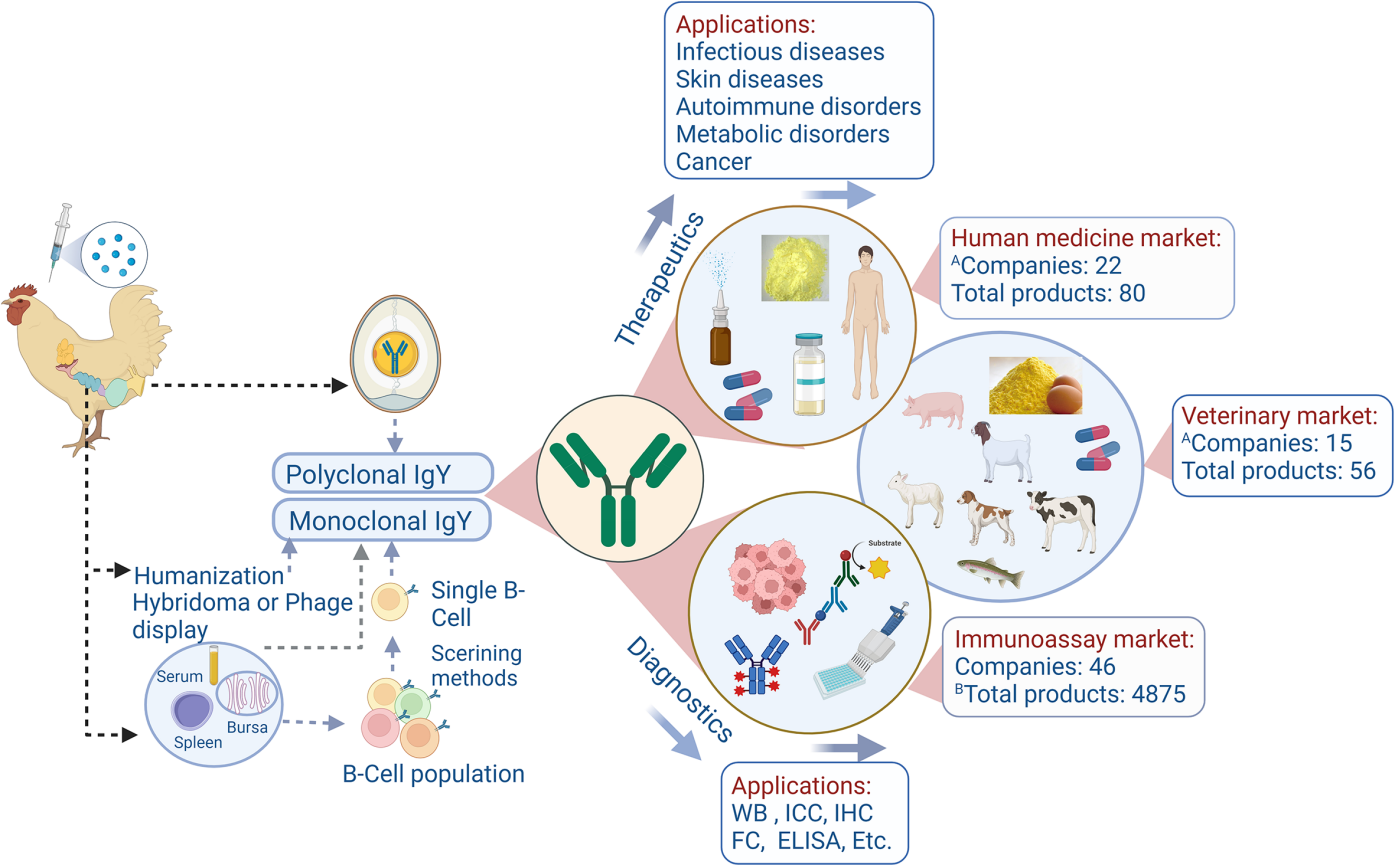
Schematic indication of the IgY production, applications, and market status. **(A)** Number of companies manufacturing IgY-based products for human medicine and veterinary fields. **(B)** Indicated the primary antibody (pAb), secondary antibody (sAb), and other products (monoclonal antibody, tag antibody, and diagnostic kit). ELISA, enzyme-linked immunosorbent assay; IC, immunocytochemistry; IHC, immunohistochemistry; FC, flowcytometry; WB, western blot. This drawing was created with BioRender.com.

IgY technology, which refers to the process involved in generating avian egg yolk antibody (IgY) with unique structural and functional properties against targeted antigens, is cost-effective, non-invasive for animal welfare, easy-to-produce, and high-volume antibody production platforms. This technology has advanced rapidly in the past decade, both in terms of technical aspects and research and clinical use (1), with broad diagnostic, prophylactic, and therapeutic applications in human and veterinary medicine. Moreover, a range of IgY-based products has entered the commercial market, attracting many investments from industries for IgY technology commercialization (2, 3) (**Figure 1**). Therefore, for the commercialization of this technology to add value to IgY-based studies, there is an urgent prerequisite for understanding the latest status of IgY-related patents, companies involved, products in the pipeline and products already marketed, marketing strategies for IgY products against conventional antibodies, regulations by product registration authorities, and addressing the difficulties and limitations for further developments in this huge arena of opportunities.

## IgY antibody: Molecular and functional aspects

IgY antibody is the predominant immunoglobulin found in the serum and egg yolk of avian, amphibian, and reptile species (2). This molecule is known as a mammalian immunoglobulin G (IgG) homolog, with a higher molecular weight (180 kDa rather than 150 kDa). Unlike IgG, which possesses three constant domains in the heavy chain, IgY contains four heavy-chain constant domains, and the hinge region in the IgY molecule is reportedly less developed than that in IgG (4, 5). The Fc region of IgY includes two carbohydrate side chains, unlike IgG, which only has one chain (2, 5). IgY has unique structural and molecular features in immunological and antibody studies. IgY is more heavily glycosylated than mammalian IgG and has significant effects on protein stability, sensitivity to proteases, immunogenicity, and biological activity (6). IgY contains complex glycans with or without core fucose (7), high mannose, and high sialic acid content; therefore, it is more stable *in vitro* and *in vivo* (7). Moreover, owing to the phylogenetic distance between avian and mammalian species, IgY has high efficacy in the recognition of proteins or epitopes that are highly conserved in mammals (5). Meanwhile, because of high ortholog protein sequence homology between mammals, IgY has less cross-reactivity than IgG in mammalian systems, that is, no interference to the mammalian Fc gamma receptors (FcγRs) and rheumatoid factors were observed, suggesting IgY as a potentially promising candidate for immunoglobulin-based therapies and immunoassays (2, 4, 5). The inherent defects in polyclonal IgY limit its potential application (4). In the last decade, the generation of monoclonal IgY or IgY fragments has made successful progress; thus, increasing functional IgY fragments, such as single chain (scFv) (8), chimeric (9), and humanized IgY (10), have been constructed. Therefore, future work on chicken monoclonal IgYs (mIgYs) should be combined with humanized techniques to avoid IgY immunogenicity. Moreover, it has been proved that hybridoma technology is not successful in monoclonal IgY production (9, 11). However, qualified functional IgY fragments, such as IgY-scFv, can be generated by using genetic engineering technology (9). A recent pilot study demonstrated that mimetics derived from IgY-scFv are feasible for both detection and therapy (11). Different independent research groups have demonstrated that generating IgY-scFv using phage display techniques is as easy as mammalian IgG-scFv generation (6, 9). Another study confirmed the therapeutic efficacy of a humanized IgY antibody against IL-12 (12). Compared with functional IgY fragments, full-length mIgY may still be valuable as an immunological tool, because it is more stable than IgY fragments (13). It is also rational to speculate that IgY may have broader functions than currently understood. Hence, a systematic IgY study may be necessary to better explore IgY molecules for IgY-scFv and mIgY applications.

## Analysis of IgY patents

**Figure 2:**
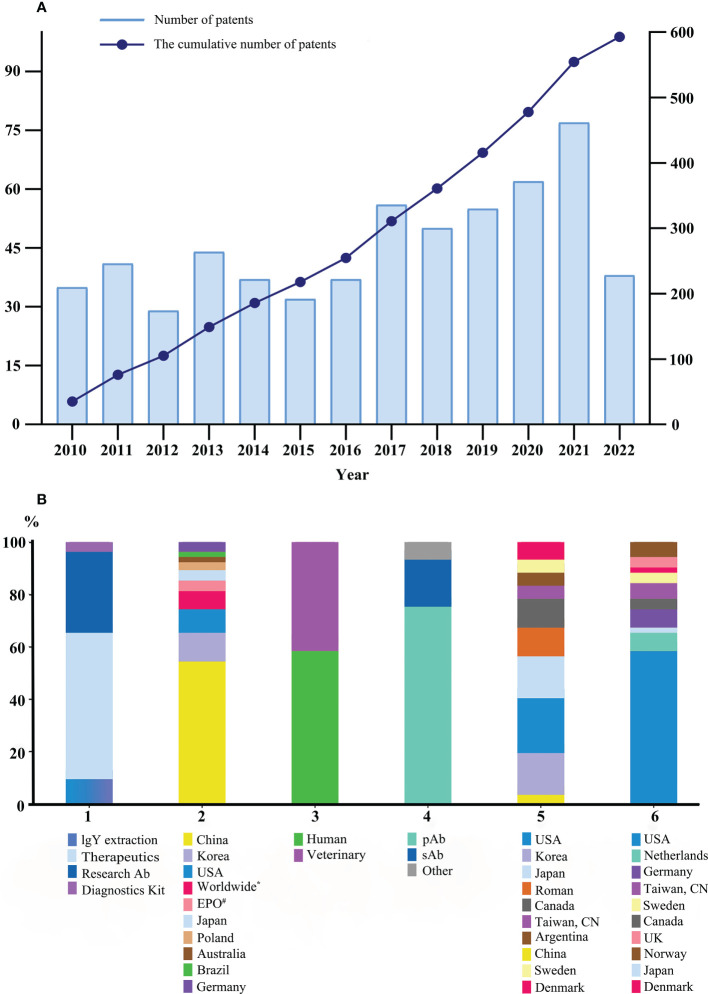
Status of IgY commercialization. **(A)** Trends in IgY patent applications, data from 2010 to June 2022. **(B)** 1: Distribution and purposes of patents applied worldwide. **(B)** 2: Geographic origins of patents related to IgY technology. Data from 2010 to June 2022. **(B)** 3: Distribution of biotherapeutics IgY product types. **(B)** 4: Distribution of diagnostic IgY (pAb: primary antibody; sAb: secondary antibody; other products: monoclonal antibody; tag antibody; and diagnostic kit). **(B)** 5 and 6: Top 10 countries for biotherapeutics and diagnostic IgY companies in the market, respectively. * Patent cooperation treaty system. # European Patent Office. Additional data on IgY commercialization in China were searched in the Chinese databanks and are summarized in **Supplementary Table S8**.

A total of 819 IgY-related patent applications were recorded in the Patentscope database (https://patentscope.wipo.int) from 2010 to 2022. The data visualization showed an increasing trend in patent applications, with the highest of 77 applications during the year 2021 (**Figure 2A**). Four broad domains were associated with the use of IgY in the patent applications field: therapeutics and prophylactic (56%), research reagent as the primary or secondary antibody (31%), IgY extraction and purification methods (11%), and diagnostic kits (2%) (**Figure 2B, 1**). IgY patent applications were mainly from China (56%), South Korea (11%), and the USA (9%), with 423, 85, and 64 patents, respectively (**Figure 2B, 2**). Most IgY patents in medical and clinical applications have been highlighted for oral or topical adjunctive use, covering broad medical needs, including oral diseases (periodontitis, gingivitis, and dental caries), gastrointestinal diseases (gastric ulcers, intestinal infectious diseases, symbiosis, toxins, and nutritional and metabolic diseases), neoplasms, and skin diseases (acne). Some IgY patents were also filed for parenteral administration in animal models (4, 14). The filing of IgY patents for diagnostic purposes, especially research antibodies, also accounts for a significant share. Due to the higher phylogenetic distance between birds and mammals, IgY has less cross-reactivity with mammalian proteins other than immunogens. Therefore, the antibodies do not bind to IgG Fc receptors and cause less false-positive staining in immunoassay studies (2, 5).

## Analysis of IgY products and market status

To analyze IgY product data, a web search was done in Google databases on the IgY products worldwide during 2010–2022 by using the keywords, “IgY product”, “IgY antibody”, and “IgY egg yolk powder supplement” and further analyzed based on two categories: biotherapeutics and diagnostic. In the biotherapeutics section, the number of IgY products for human medicine was higher than for veterinary products (59% vs. 41%; **Figure 2B, 3**). In human medicine, 80 products were identified in different phases of development as products on the market (52), under discovery (13), in preclinical studies (7), in pipelines (7), and clinical trials (1) (**Figure 1** and **Supplementary Table S1**). In the veterinary field, 56 products were identified as available at various stages, including products in the market (54), discovery (2), and development (1) (**Figure 1** and **Supplementary Table S2**). In the diagnostic sector, 4875 products were introduced into the market: 3729 as primary antibodies, 846 as secondary antibodies, and 253 as other products (**Supplementary Table S3**). IgY can be used as a natural and cost-effective molecule providing passive immune protection against various pathogens including bacteria, viruses, fungy, and parasites in humans and animals (15), which includes the products recommended for oral cavity infections such as Ig-Guard Mutant and Ovalgen^®^ DC (*Streptococcus mutans*), Ovalgen^®^ PG (*Porphyromonas gingivalis*), and Ovalgen^®^ CA (*Candida albicans*), for respiratory infections, such as Ovalgen^®^ FL (influenza) and anti-SARS-CoV-2 IgY, for gastrointestinal diseases, such as Ig-Guard Helico, GastimunHP, Ovalgen^®^ HP (*Helicobacter pylori*), Ig-Guard Rota, Ovalgen^®^ RV (Rotavirus), IgY Max (against 26 human-relevant bacteria) and products that are in discovery stage (IGY-106, IGY-112, IGY-108, and PG-001), for skin disorders, such as Ig-Guard Acne (*Propionibacterium acnes*) and ImmunoDerm Y (*Staphylococcus epidermidis*), for hypercholesterolemia, such as Ig-Guard cholesterol (against lipase and amylase), for balancing and improving the immune system, such as Vector450 and Ovopron^®^ (Yolk powder contain non-specific polyclonal IgY), for treating hair loss, such as OstriGrow (contains IgY against dihydrotestosterone) (2–4). As most of these diseases are caused by multiple pathogens, IgY intervention has the advantage of “cocktail therapy” by targeting multiple pathogens, and a wide array of therapies can be easily established and precise treatment can be provided based on the disease etiology analysis and patient’s health conditions (15). In the veterinary and animal fields, there are many IgY products such as Ig-Guard Calf, Ig Lock Calves, Globigen^®^ Dia Stop, and IgY DNT (for calf diarrhea); PG-002 (for cow mastitis); Ig-Guard Swine, Ig Lock Pig (for swine diarrhea), Ig-Guard Puppy, ParvoONE^®^, Ig Lock Canine, GastroMate^®^, and Guardizen (for pets, especially in canines); Ig-Guard Duck, Ig-Guard Poultry, BIOAb DHV-IgY (for poultry), Ig-Guard (SH), Ig-Guard (SA), Ig-Guard (AE), Ig-Guard (AY), and PG-003 (for aquaculture) (2–4). In the diagnostic sector, primary antibodies represented 77% of these products, followed by secondary antibodies (18%), and other products (5%) (**Figure 2B, 4**). The availability of IgY products for diagnostic use in the market can be related to the unique IgY characteristics mentioned above, with less background noise and lower IgY aggregation than mammalian IgG (16, 17). Monoclonal IgY application is yet another growing market segment, although most products are still in the discovery or development stages, in which an anti-PD1 humanized IgY, Sym021, has been registered for clinical trials.

## Analysis of IgY manufacturing industries

Information on IgY manufacturing companies was retrieved from the Google database by using the keywords, “IgY company” and “IgY product data” worldwide from 2010 to 2022, and information about 73 companies from their respective websites was analyzed in relation to their IgY products (**Supplementary Tables S4–S6**). Notably, more companies were working in the field of diagnostics, including primary and secondary antibodies for research purposes, than biotherapeutics (46 companies against 27). Over 95% of IgY products produced by these companies are polyclonal antibodies. Regarding the companies producing biotherapeutics products, the USA (21%), South Korea (16%), Japan (16%), and Romania (11%) had the largest percentages, with four, four, three, and two companies, respectively (**Figure 2B, 5**). In the diagnostic sector, the USA represented 60% of the companies (**Figure 2B, 6**). Most diagnostic companies were manufacturers, except for three distributors. In the biotherapeutic sector, companies focus on human and veterinary medicine products, which are often polyclonal antibodies against pathogens, such as dietary supplements, animal feed, and topical use. Some manufacturers included AD Biotech Co, DAN Biotech Inc., IgY-research, Imunoinstant, Good Biotech Corp, IgY Nutrition, EW Nutrition, IGY Life Sciences Inc., Xymogen, Prnpharmacal, Pharma Foods International, Bioinnovo, Vetglory, Immunsystem, and Eggcellent Proteins (**Supplementary Table S4**). Polyclonal IgY antibodies have been available in the market for over two decades (15). Contrary to the common understanding of antibody products, IgY is also referred to as GRAS (generally recognized as safe) by the US Food and Drug Administration (FDA), and regulations in licensing IgY products are relaxed, especially as food supplements or for oral use (15). However, there exist strict regulations for the parenteral administration products and monoclonal IgY (18, 19). As technology advances, many companies have placed functional IgY fragment products on their agenda for future registration and commercialization, as well as a platform to provide antibody production services (1, 2). For example, OmniAb Company offers humanized antibody technology (OmniChicken^®^), which claims the production of fully humanized antibodies based on genetically engineered chickens that express human immunoglobulin assemblies (20). In the diagnostic sector, most companies are manufacturers of research antibodies and the main producers include Usbio, Lsbio, Genetex, OriGene Technologies Inc., Abcam, Sigma-Aldrich, Exalpha, Abnova, Agrisera, and Dianova (**Supplementary Table S5**). To date, there is no comprehensive report on the IgY products market, including the number of sales, product type, working capital, and market value of the companies. Nevertheless, analysts have shown that the IgY market, especially polyclonal products in the research sector (primary or secondary antibodies) will grow significantly by 2027. The market value of IgY polyclonal antibodies is poised to USD 14.2 million by 2027 ending at a CAGR (compound annual growth rate) of over 13.4% during the forecast period of 2020 to 2027. However, the total antibody market share is only 0.24% (3).

## Analysis of registration and approval of IgY products

To analyze licensed IgY products, clinical trial databases were analyzed on IgY products worldwide from the year 2005–2022 to using the keywords, “IgY” (clinicaltrials.gov). A total of 15 clinical trials were recorded, of which 12 were in the study phase and 3 were commercialized (**Supplementary Table S7** worldwide and **Supplementary Table S9** in the Chinese databank). The first IgY clinical trial was a gargle solution containing specific IgY antibodies against *Pseudomonas aeruginosa* for daily administration in patients with cystic fibrosis registered in 2011 (Identifier (ID): NCT01455675). In another clinical trial, the efficacy and safety of the food supplement IGN-ES001 (egg yolk powder containing a specific IgY against *Escherichia coli* F18ab and *Salmonella typhimurium*) were demonstrated (ID: NCT03058224). A study showed that dietary supplements containing specific IgY against *Helicobacter pylori* (GastimunHP) reduced bacterial colonization and pathogenicity in patients with gastric ulcer (ID: NCT02721355). In children with diarrhea, using IgY-based nutritional products reduced the duration and severity of diarrhea (ID: NCT02385773). Daily oral administration of an IgY product (IgY Max^®^) containing a mixture of IgY antibodies against 26 human-relevant bacteria improved intestinal integrity and reduced microbiome imbalance (ID: NCT02972463). The use of an oral IgY-specific drug (IM-01) reduced the growth of *C. difficile* pathogen (ID: NCT04121169). A clinical trial using IgY (dried yolk powder diet supplement) reduced intestinal cholesterol uptake and regulated serum cholesterol levels (ID: NCT01890889). IgY (AGY-010) capsules against celiac disease (CD) reduce immunosuppression and disease complications (ID: NCT03707730). Furthermore, a diet supplement (egg lozenge) containing a specific IgY against *Porphyromonas gingivalis* reduced the number of pathogens in oral infections (ID: NCT02705885). In another experiment on dental caries, the use of a specific IgY against *Streptococcus mutans* inhibited bacterial growth (ID: NCT02341352). Recently, for coronavirus infection, a clinical trial using anti-SARS-CoV-2 IgY in the form of a nasal spray reduced viral pathogenicity (ID: NCT04567810). According to our investigation, only one clinical trial has been confirmed in the parenteral administration of IgY product (ID: NCT03311412); namely, monoclonal IgY product (Sym021) against human programmed cell death protein 1 (PD1) showed promising inhibitory binding to PD-1 (21).

## Industrialization of IgY technology: Challenges and prospective solutions

### Safety of IgY products

Immunogenic responses after polyclonal IgY administration have been described *via* oral (22) and parenteral routes (23). Recently, the safety of anti-SARS-CoV-2 IgY for nasal drop administration was confirmed with lack of cross-reactivity with human lung and nasal mucosa tissues and adverse events in a phase 1 clinical study (24). In another nasal delivery study, the protective effect and safety of anti-SARS-CoV-2 IgY were confirmed in a mouse model, with no adverse effects observed (25). Moreover, intraperitoneal injection of polyvalent-specific IgY against Zika virus in a mouse model, besides not inducing antibody-dependent enhancement (ADE), did not display any side effects (26). Despite the lack of cross-reactivity between IgY and mammalian Fc, further safety studies are needed for systemic administration. The development of chimeric antibody fragments and humanized monoclonal IgY such as Sym021 could also contribute to reducing these safety concerns (21).

### Large scale production of IgY antibodies

IgY technology requires only a low cost for large-scale production but with high-volume production. This aspect is evident from the fact that one hen can produce approximately 325 eggs with 50–100 mg/egg IgY, thus yielding up to 35 g of IgY annually (27), which is five times higher in specific pathogen-free (SPF) hens (175 g vs. 35 g) (2). Standard laboratory techniques for IgY extraction usually include precipitation methods using salts (e.g., ammonium sulfate), polymers (e.g., polyethylene glycol), and affinity chromatography techniques for purification (28). However, these methods can be time-consuming or expensive (15, 28), which may interfere with their large-scale application. The extraction of egg components at an industrial scale can be achieved using membrane techniques or powder processing. For example, ultrafiltration (UF), microfiltration, and reverse osmosis have been successfully implemented to concentrate and/or extract egg components (24, 26). However, only a few studies have standardized the use of UF membrane processes to purify IgY antibodies. Lipids or lipoproteins can strongly influence the efficiency of UF membranes; thus, their removal from the water-soluble fraction remains a critical factor in the success of this method (25, 28). Moreover, some commercial IgY extraction and purification kits are available on the market, but they are not suitable for industrial-scale manufacturing processes. Except for the spray drying method (egg yolk powder preparation as IgY product), the available variety of IgY extraction methods and purification methods discussed above have not been applied well in large-scale production processes. Therefore, IgY extraction and purification standardization and optimization on an industrial scale are required.

### Stability of IgY products

It is crucial to maintain the stability of the product and, thus, the bioactivity of IgY under different storage and usage conditions. IgY is more hydrophobic with high stability, retains its activity for 6 months at room temperature and 5 years at 4°C, is relatively stable up to 70°C for 15 min, and is highly active at pH 4–11 (5, 15). Moreover, IgY can be degraded similarly to IgG by digestive enzymes, such as proteases and hydrolases (15). Few studies have been conducted on the half-life of IgY in the body. For example, active IgY is present in the patient’s saliva 12 h after gargling (29). In newborn pigs, IgY has a half-life of 1.85 d in the sera and 1.73 h in the gastrointestinal tract (30). Therefore, efforts are needed to identify the most suitable protective agent, carrier, and proper dosage design and delivery systems to retain IgY product stability.

### IgY delivery systems and dosage form designs

Few studies have been conducted on the IgY delivery systems and dosage forms compared with mammalian IgG. Some IgY properties pose a limiting factor for the delivery method. For example, a lower isoelectric point (IP: 5.7–7.6) than IgG (IP: 6.1–8.5), causes no efficient fluid-phase endocytosis (pinocytosis) in mammalian cells (31). Moreover, IgY has a shorter half-life than IgG in the mammalian host, as IgY-Fc cannot bind to mammalian FcγRs (32). Therefore, it can affect the choice of IgY delivery system or dosage form (32). Different forms of mIgYs for systemic administration are in pipelines, such as the humanized IgY platforms OmniChicken^®^ (20) and Sym021^®^ (21). However, extensive research, especially on functional IgY-scFv fragments (33), is highly limited. However, strict regulations exist for parenterally administered products and monoclonal IgY, and there are problems with effective delivery systems. The use of egg yolk powder containing specific IgY as a food ingredient is an economical and practical “dosage form” for the oral administration of polyclonal IgY along with encapsulation materials and stabilizers (15, 28). Attempts have been made to deliver the IgY active ingredient to the oral cavity (29) and nasal cavity (24) as parenteral administrations, as well as systemic administration of mIgY such as Sym021. However, in-depth scientific research is still awaited for the safety approval and efficacy of the IgY dosage form design.

### Regulatory mandates for IgY products commercialization

Food and drug regulators, such as the FDA, play a key role in the introduction of novel products into the market. IgY products are available in the market for oral administration, as listed (**Supplementary Tables S1 and S2**). This approval has been favored by regional regulations that allow the registration of biological products as functional foods (34). The development of IgY-based compositions should also focus on key production aspects, such as close biological activity monitoring, the presence of contaminants, and quality control parameters (34). Compositions designed for oral delivery may be easier to register than IgY-based products, which require parenteral administration. Thus, more studies on the safety and efficacy of IgY delivery *via* this route are still needed. For example, it is required to have standards such as good manufacturing practice (GMP) conditions, specific-pathogen-free (SPF) birds, and IgY safety documentation for industrialization.

## Conclusion

Quantitative IgY technology analysis reveals significant progress evident from patents, companies, clinical trials, and products in diagnostic and biotherapeutic fields for human and animal applications. In the diagnostic field, owing to the unique properties of IgY over mammalian IgG discussed above, IgY-based immunoassay products are better developed in the market than biotherapeutics. Generally, IgY biotherapeutic products are marketed as oral or topical supplements. Despite the range of these products entering the market, parenteral IgY products as licensed drugs have made little progress, probably because of the lack of standards in experimental animals (i.e., specific-pathogen-free birds) and technical processes (i.e., validated and diversified extraction and purification methods), as well as the consensus on regulation and approval of IgY-based health products. There is only one monoclonal IgY product in the clinical phase, but some products are in development, indicating the emerging application of mIgY and functional IgY fragments as novel drug candidates for the utilization of IgY antibodies. Finally, studying and understanding the industrialization and commercialization status of the IgY technology is essential for future development.

## Author contributions

SY and XZ contributed to conception and design of the study. SY and RW organized the database. SY performed the statistical analysis. SY wrote the first draft of the manuscript. SY, RW, and BC wrote sections of the manuscript. All authors contributed to manuscript revision, read, and approved the submitted version.

## Funding

This work was supported by the National Natural Science Foundation of China [grant number 31873006].

## Conflict of interest

The authors declare that the research was conducted in the absence of any commercial or financial relationships that could be construed as a potential conflict of interest.

## Publisher’s note

All claims expressed in this article are solely those of the authors and do not necessarily represent those of their affiliated organizations, or those of the publisher, the editors and the reviewers. Any product that may be evaluated in this article, or claim that may be made by its manufacturer, is not guaranteed or endorsed by the publisher.
