# Supplementary material for: Trends in industrialization and commercialization of IgY technology
Source: Front Immunol. 2022 Oct 20;13:991931. doi: 10.3389/fimmu.2022.991931 (PMC9630564; doi:10.3389/fimmu.2022.991931)
Supplement: Supplementary file 1 [file DataSheet_1.docx]

**Supplementary Data**

**Trends in Industrialization and Commercialization of
IgY Technology**

**Supplementary Tables**

**Supplementary Table S1. IgY human medicine products for biotherapeutics worldwide***

| Human medicine/ healthy/ food supplements | | | |
| --- | --- | --- | --- |
| IgY Targets/Pathogens | Application/ Description | No. of products | Phase |
| *Helicobacter pylori* | Anti-Ulcers disease  Anti-Ulcers disease | 4 | In Market |
|  |  | 1 | Discovery |
| *Helicobacter pylori, Enterovirus, Rotavirus* | Gastrointestinal (GI) infections | 1 | Development |
| Lipase and Amylase | Anti-Obesity and cholesterol | 1 | In Market |
|  | Anti-Obesity and cholesterol | 1 | Development |
| *Candida albicans* | Anti-*Candida* IgY  Anti-*Candida* IgY | 2 | In Market |
|  |  | 1 | Preclinical |
| *Citrobacter* | Anti *Citrobacter* IgY | 1 | In Market |
| *Clostridium difficile* | Anti-*Clostridium difficile* IgY | 4 | In Market |
|  |  | 1 | Discovery |
| *Enterococcus faecalis* | Anti-*Enterococcus faecalis* IgY | 1 | In Market |
| *Escherichia coli* | Anti-*Escherichia coli* IgY | 1 | In Market |
| *Klebsiella pneumoniae* | Anti-*Klebsiella pneumoniae* | 1 | In Market |
| *Proteus mirabilis* | Anti *Proteus mirabilis* IgY | 1 | In Market |
| *Pseudomonas aeruginosa* | Cystic Fibrosis | 2 | In Market |
|  | Cystic Fibrosis | 1 | Preclinical |
| *Salmonella enteritidis* | Anti-*Salmonella enteritidis* IgY | 1 | In Market |
| *Staphylococcus aureus* | Anti-*Staphylococcus aureus* | 2 | In Market |
| *Staphylococcus epidermidis* | Anti-*Staphylococcus* IgY | 1 | In Market |
| *Streptococcus pneumonia* | Anti-*Streptococcus pneumoniae* | 1 | In Market |
| Borelioza | Anti Borelioza IgY | 1 | In Market |
| *Herpes simplex* virus | Anti-*Herpes* IgY | 1 | In Market |
| Human *Papillomavirus* | Anti-HPV IgY | 1 | In Market |
| Virus *Varicelo*-*Zosterian* | Anti *Varicelo*-*Zosterian* IgY | 1 | In Market |
| Human *Rotavirus* | Anti-*Rotavirus* IgY  Anti-*Rotavirus* IgY  Anti-*Rotavirus* IgY | 3 | In Market |
|  |  | 1 | Preclinical |
|  |  | 1 | Discovery |
| *Pseudomonas aeruginosa, Klebsiella pneumoniae, Salmonella spp., Escherichia coli, Enterococcus faecalis, Salmonella enteritidis, Salmonella typhimurium, Streptococcus mutans, Staphylococcus aureus, Streptococcus group B, Proteus mirabilis, Acinetobacter baumannii, Helicobacter pylori, Clostridium difficile-bacterial bodies, Clostridium difficile-anatoxin, Candida albicans, Candida glabrata, Candida krusei* | Multiple for Gastrointestinal (GI) infections | 1 | In Market |
| *E.Coli, E.Coli (Aerobacter),Klebsiella pheumoniae, Salmonella (Typhimurium, Enteriditis, Simulans, Dysenteriae, Epidermis), Streptococcus pyogenes* (Type 1,3,5,8,12,14,18,22), *Streptococcus ( Agalactiae, Mulans, Mitis, Salavarius, pneumoniae, Sanguis), Propionibacterium acnes, Haemophilis influenza, Pseudomonas ( Aeruginosa, Vulgaris)* | Multiple for Gastrointestinal (GI) infections | 1 | In Market |
| *Streptococcus mutans* | Prevents dental caries  Prevents dental caries  Prevents dental caries | 2 | In Market |
|  |  | 1 | Clinical |
|  |  | 1 | Development |
| *Propionibacterium acnes* | Protects and prevents acne  Protects and prevents acne | 1 | In Market |
|  |  | 1 | Discovery |
| *Porphyromonas gingivalis* | Anti-*Porphyromonas gingivalis*  Anti-*Porphyromonas gingivalis* | 1 | In Market |
|  |  | 1 | Clinical |
| *Influenza virus* ( H1N1, H2N2, H3N2, and H5N1 | Anti-*Influenza* IgY for seasonal flu  Anti-*Influenza* IgY for seasonal flu | 1 | Preclinical |
|  |  | 1 | Discovery |
| *Cronobacter sakazakii* | Anti-*Cronobacter sakazakii*IgY for infant milk formula | 1 | In Market |
| COVID-19 | Anti- SARS-CoV-2 IgY | 2 | Preclinical |
| Irritable Bowel Disease | Anti-Irritable Bowel Disease IgY | 1 | Discovery |
| African Swine *Flu* | Anti-African Swine *Flu* IgY | 1 | Development |
| *Clostridium difficile, Vibrio cholera, E. coli, Salmonella, Shigella, and norovirus* | Gastrointestinal diseases | 1 | In Market |
| Dihydrotestosterone (DHT) | Helps to prevent hair loss | 1 | Preclinical |
| *Acinetobacter baumannii A* | Anti *Acinetobacter baumannii A* | 1 | In Market |
| *Norovirus* | *Norovirus*  Human prophylactic | 1 | Discovery |
| Unknown | Non-specific IgY antibody | 1 | In Market |
| Vector450 | Non-specific IgY antibody extracted from antibiotic-free eggs | 1 | In Market |
| Human immunoglobulin | Genetically engineered chickens expressing human immunoglobulin repertoires | 1 | Development |
| Programmed cell death protein 1 (PD1) | Anti-PD1 mAb | 1 | Discovery |
| G-protein-coupled receptors (GPCRs) | Anti- GPCRs mAb | 1 | Discovery |
| Neurotensin receptor 1 (NTSR1) | Anti- NTSR1 mAb | 1 | Discovery |
| Bradykinin receptor B1 (BKRB1) | Anti- BKRB1 mAb | 1 | Discovery |
| Clostridium difficile | Anti- C. difficile mAb | 1 | Discovery |
| Purinergic receptor P2Y6 | Anti- Purinergic receptor P2Y6 mAb | 1 | Discovery |

*Due to the data ownership, the names of the products and companies have not been provided

**Supplementary Table S2. IgY veterinary (pet and livestock) medicine products for biotherapeutics worldwide***

| Veterinary medicine/ healthy/ feed supplements | | | | |
| --- | --- | --- | --- | --- |
| IgY Targets/Pathogens | Application/Description | Species | No. of products | Phase |
| Bovine *Rotavirus* (Serotypes G6 and G10), Bovine *Coronavirus, Salmonella typhimurium, Salmonella Dublin, Escherichia coli K99, Cryptosporidium parvum, Clostridium perfringens* | Calf diarrhoea | Calf | 5 | In Market |
| *Escherichia coli* strans (K88, 987P, K99), *Clostridium perfringens*, *Salmonella typhimurium*, Porcine *Rotavirus*, Transmissible gastroenteritis virus (TGEV), Porcine epidemic diarrhea virus (PEDV), PRV(porcine rotavirus) | Piglet diarrhea | Piglet | 4 | In Market |
|  |  |  | 1 | Development |
| Canine *distemper* virus, infectious canine hepatitis virus, Canine *parvovirus*, Canine *parainfluenza* virus, and Canine *corona* virus, Canine *adenovirus*, Canine *rotavirus* | Canine disease | Canine | 5 | In Market |
| *Colibacillosis*, *Salmonellosis* | Poultry Diarrhea | Poultry | 1 | In Market |
| Pericardial effusion-hepatitis syndrome (HHS), *Adenovirus*, H9 avian *influenza*, *Newcastle* disease (ND) Infectious bursal diseases (IBD) ) | Poultry disease | Poultry | 1 | In Market |
| *Staphylococcus aureus*, coagulase-negative *staphylococcus*, *Streptococcus uberis, Streptococcus dyslactiae, Streptococcus agalactiae, enterococci* and *Escherichia coli* | Mastitis mitigation in milking cows | Dairy cows | 2 | Discovery |
| Hepatopancreatic Acute Necrosis Syndrome | Early Mortality Syndrome | Shrimp | 1 | In Market |
| Salmonid Rickettsial Septicaemia | Salmon disease | Salmon | 2 | In Market |
| Aquaculture White Spot Syndrome Virus (WSSV) | White Spot disease | Fish | 1 | Discovery |

*Due to the data ownership, the names of the products and companies have not been provided

**Supplementary Table S3. The main IgY diagnostic products in the worldwide market***

| Primary Antibody (pAb) |  |
| --- | --- |
| Products | Application |
| Anti-N-Acetylglucosamine Kinase (NAGK) | E, WB |
| Anti-Ciliary Neurotropic Factor (CNTF) | E, IHC, WB |
| Anti-c-Myc | E, WB |
| Anti-GAPDH, NT | E, WB |
| Anti-Fox3 | IC, IHC |
| Anti-Prothrombin | WB |
| Anti-Pro-Platelet Basic Protein (PPBP) | WB |
| Anti-Thyroid Stimulating Hormone, beta (TSHb) | E |
| Anti-Protein Phosphatase 1 gamma 2 (PP1g2) | E |
| Anti-ErbB2 | E, IF, IHC, IP, WB |
| Anti-WDR40A | E, IC, IF, WB |
| Anti-Protein A | E |
| Anti-Protein Phosphatase 1 beta (PP1b) | E |
| Anti-Elongin C | E |
| Anti-Aquaporin 6 (WCH3, hKID, AQP2-L) | E |
| Anti-Angiotensin II Type 2 Receptor (AT2) | E, WB |
| Nestin Chicken anti-Human pAb | IC, IHC-P, WB |
| Vimentin Chicken anti-Human pAb | IHC, IHC-P |
| MAP2 Chicken anti-Human pAb (C-Terminus) | IHC, IHC-P |
| GFAP Chicken anti-Human pAb | IC, IHC, WB |
| Tubulin Beta 3 Chicken anti-Human pAb | IC, IHC-P, WB |
| CK1 Alpha Chicken anti-Human pAb | IC, IF, IHC-, WB |
| PGP9.5 Chicken anti-Human pAb | IC, IF, WB |
| BDNF Chicken anti-Human pAb | E, WB |
| LINE-1 Chicken anti-Human pAb | E, WB |
| beta Tubulin 3/ Tuj1 antibody | WB, IC/IF, IHC |
| AKT antibody | WB |
| HEY1 antibody | WB, E |
| Albumin antibody | WB, IHC, E |
| Anti-GFP antibody | IC/IF, WB |
| Anti-Neurofilament heavy polypeptide antibody | IC, IHC-Fr, WB |
| Anti-NeuN antibody - Neuronal Marker | IHC-Fr, & P, WB |
| Anti-Tyrosine Hydroxylase antibody | IHC-FrFl, IHC-P |
| Anti-Tau antibody | IC/IF, IHC-P, WB |
| Anti-GAD67 antibody | IC/IF, IHC-P |
| Anti-mCherry antibody | IC/IF, WB |
| lacZ Chicken pAb | IF, IHC, WB |
| CD11b (ITGAM) Chicken pAb | IF, IHC |
| MRP8/14 (S100A8/A9) Chicken pAb | E, IHC |
| alpha Tubulin (TUBA1A) Chicken pAb | WB |
| Nes Chicken Polyclonal Antibody | IF, IHC, WB |
| beta Actin (ACTB) Chicken Polyclonal Antibody | IF, WB |
| Chicken HexaHistidine (6His) | E,WB |
| Chicken anti Human placenta-derived cytonectin | E, WB |
| Chicken anti Human Haemoglobin | E, WB |
| Chicken anti Mycobacterium MPB83 | E, WB |
| anti-Bacillus anthracis Lethal Factor A, (Biotin) | E, WB |
| Chicken anti Protein G Antibody | E, WB |
| Polyclonal (IgY) to Human ANKLE1 | E, WB |
| Polyclonal (IgY) to Human DUT / DUTPase | WB, IHC |
| Chicken Polyclonal (IgY) to Human EMX1 | WB, IHC, E |
| Polyclonal (IgY) to Human NF-L / NEFL | WB, IHC, E |
| Polyclonal (IgY) to Human SDHAF2 | WB, IHC, E |
| Polyclonal (IgY) to Human TMPRSS11A | WB, IHC, E, IC |
| Polyclonal (IgY) to Human VILIP / VSNL1 | IHC, IC |
| Chicken Polyclonal to Human KLHL25 | WB, IHC, E |
| Acpp polyclonal antibody | IHC, IC |
| Beta-Galactosidase polyclonal antibody | WB,IHC,IC |
| BrdU polyclonal antibody | IHC,E |
| Clostridium difficile Toxin A pAb | WB,E |
| CORO1A polyclonal antibody | IHC, IC |
| CHAT polyclonal antibody | IHC, IC |
| Secondary Antibody (sAb) |  |
| Chicken anti-Human IgG Fc | E, WB |
| Chicken anti-Dog IgG (H+L) | E, WB |
| Chicken anti-Human IgG (H/L) | E, WB |
| Chicken anti-Human IgG (Fab)2 | E, WB |
| Chicken anti-Mouse IgG (H+L) | E, WB |
| Chicken anti Rabbit IgG (H+L) | E, WB |
| Human IgM Chicken anti-Human pAb | E, WB |
| Chicken Anti-Rabbit IgG antibody (Biotin) | WB, E, IC, IHC |
| Chicken Anti-Human IgG antibody (HRP) | WB, E, IC, IHC |
| Chicken Anti-Human IgG antibody (AP) | WB, E, IC, IHC |
| Chicken Anti-Mouse IgG antibody (Rhodamine) | IC, FACS, E |
| Chicken Anti-Mouse IgG antibody (Biotin) | WB, E, IC, IHC |
| Chicken Anti-Mouse IgG antibody (HRP) | WB, E, IC, IHC |
| HRP Anti-HA tag antibody | E, WB |
| FITC Anti-Myc tag antibody | Flo, WB |
| Biotin Anti-Protein A antibody | E, WB |
| HRP Anti-Digoxigenin antibody | E, WB |
| HRP Anti-6X His tag® antibody | E, WB |
| HRP Anti-Protein A antibody | E |
| Biotin Anti-LDL antibody | E, RIA |
| FITC Anti-6X His tag® antibody | E, WB |
| HRP Anti-Hemagglutinin antibody | E, WB |
| HRP Anti-Protein G antibody | E, WB |
| Chicken anti-Goat IgG | WB, IHC, E |
| Chicken anti-Goat IgG (HRP) | WB, E |
| Chicken anti-Human IgG (FITC) | IHC, IC |
| Chicken anti-Mouse IgG (FITC) | IHC, IC |
| Chicken Anti-Rat IgG Antibody (AP) | E, IHC, WB |
| Chicken Anti-Mouse IgG (Fc fragment) antibody | E, WB |
| Chicken Anti-Rabbit IgG antibody (FITC) | Flo, IHC, IC, E |
| Chicken Anti-Rat IgG antibody (Biotin) | WB, IHC, E |
| Chicken Anti-Human IgA Antibody | E, IC, IHC, WB |
| Other Antibody# |  |
| Anti-Flag (DYKDDDDK) Epitope Tag | E |
| Anti-HA Tag (Hemagglutinin) (FITC) | FLISA, IF, WB |
| Chicken IgY isotype control | FACS, E |
| Chicken IgY ELISA Kit | E |
| Chicken Polyclonal turboGFP Antibody | WB |
| Anti-DDK (FLAG) chicken polyclonal antibody | WB |
| Anti-Tdtomato chicken polyclonal antibody | WB |
| Chicken Polyclonal mPlum Antibody | WB |
| Normal Ostrich IgY (egg-derived) | E, WB |
| Normal Chicken IgY (egg-derived) | E, WB |
| Normal Goose IgY (egg-derived), (Biotin) | E, WB |
| Normal Quail IgY (egg-derived), (Biotin) | E, WB |
| Normal Duck IgY (egg-derived), (Biotin) | E, WB |
| c-Myc tag polyclonal antibody (Biotin) | WB, E |
| HRP Conjugated Chicken anti-C-MYC | WB, E, Flo |

*Due to the data ownership, the names of the products and companies have not been provided; ^#^Other Antibody: Monoclonal antibody; Tag antibody; and diagnostic kit, E: ELISA, IC: Immunocytochemistry, IHC: immunohistochemistry, WB: Western blot, FC: Flowcytometry, IF: Immunofluorescences

**Supplementary Table S4. Number of all biotherapeutics available IgY companies in the “Market” worldwide until June 2022**

| Companies | Website | Product type | Country/  Region |
| --- | --- | --- | --- |
| Xymogen | https://www.xymogen.com/ | 1 | US |
| Prodigybiotech | http://www.prodigybiotech.com/ | 1, 2 | US |
| Prnpharmacal | https://prnpharmacal.com/ | 2 | US |
| Omniab | https://www.omniab.com/ | 1 | US |
| Avianax LLC | https://www.avianax.com/ | 1 | US |
| Dongbangah | http://www.dongbangah.com/ | 2 | South Korea |
| AD Biotech Co. | http://adbiotech.com/ | 1, 2 | South Korea |
| DAN Biotech, Inc. | http://www.danbio.com/ | 1, 2 | South Korea |
| GastimunHP | https://gastimunhp.vn/ | 1 | Japan |
| IgY-research | http://www.igy-research.com/ | 1, 2 | Japan |
| Pharma Foods International | https://www.pharmafoods.co.jp/ | 1 | Japan |
| Romvac | http://romvac.ro/ | 2 | Romania |
| Imunoinstant | https://imunoinstant.romvac.ro | 1 | Romania |
| Good Biotech Corp. | http://www.good-biotech.com/ | 1 | Taiwan |
| Bioinnovo | http://bioinnovo.com.ar/ | 2 | Argentina |
| Vetglory | http://www.vetglory1.com/ | 2 | China |
| Immune Biosolutions | https://immunebiosolutions.com/ | 1 | Canada |
| IgY Nutrition | https://www.igynutrition.com/ | 1 | Canada |
| Immunsystem | http://www.immunsystem.com/ | 1 | Sweden |
| Symphogen | http://www.symphogen.com/ | 1 | Denmark |
| Ovagen Group Limited | http://www.ovagen.ie/ | 1 | Ireland |
| NABAS | http://www.nabas.no/ | 1 | Norway |
| IgYTechnology.com | https://www.igytechnology.com/ | 1 | Portugal |
| IGY Life Sciences, Inc. | http://www.igylifesciences.com/ | 1 | Canada |
| IgY Immunologix | http://www.igylx.com/ | 1, 2 | India |
| Eggcellent Proteins | https://www.eggcellentproteins.com/ | 1 | Scotland |
| EW Nutrition | https://ew-nutrition.com/ | 1, 2 | Germany |

1: Human medicine 2: Veterinary medicine.

**Supplementary Table S5. The number of all diagnostic available companies in the “Market” worldwide that have some IgY products as manufacturing or selling until June 2022**

| Manufacturing companies |  |  |  |
| --- | --- | --- | --- |
| Companies | Website | Product type | Country/  Region |
| Novus Biologicals, LLC | https://www.novusbio.com/ | 1,2,3 | US |
| Usbio | https://www.usbio.net/ | 1,2,3 | US |
| Lsbio | https://www.lsbio.com/ | 1,2 | US |
| Genetex | https://www.genetex.com/ | 1,2 | US |
| R&D Systems | https://www.rndsystems.com/ | 1,2,3 | US |
| Biolegend | https://www.biolegend.com/ | 1 | US |
| Thermo Fisher Scientific | https://www.thermofisher.com/ | 1,2 | US |
| OriGene Technologies Inc. | https://www.origene.com/ | 1,2,3 | US |
| Abcam | https://www.abcam.com/ | 1,2 | US |
| Proteus-biosciences | http://www.proteus-biosciences.com/ | 1 | US |
| Santa Cruz Biotechnology, Inc | https://www.scbt.com/ | 1,2,3 | US |
| Abbexa | https://www.abbexa.com/ | 1 | US |
| Cedarlanelabs | https://www.cedarlanelabs.com/ | 2 | US |
| ImmunologyConsultants Laboratory | http://www.icllab.com/ | 1,2,3 | US |
| Fitzgerald-fii | https://www.fitzgerald-fii.com/ | 1,2 | US |
| Sigmaaldrich | https://www.sigmaaldrich.com/ | 1,2,3 | US |
| Exalpha | https://www.exalpha.com/ | 1,2,3 | US |
| Boster Biological Technology | https://www.bosterbio.com/ | 1,3 | US |
| Aviva Systems Biology | https://www.avivasysbio.com/ | 1 | US |
| EnCor Biotechnology Inc. | https://encorbio.com/ | 1 | US |
| PhosphoSolutions | https://www.phosphosolutions.com | 1 | US |
| GenWay Biotech Inc | https://www.genwaybio.com/ | 1 | US |
| Cusabio | https://www.cusabio.com/ | 1 | US |
| Accuratechemical | http://www.accuratechemical.com/ | 1 | US |
| Bio-Genex | https://store.biogenex.com/ | 1 | US |
| American Research Products | https://www.arp.com/ | 1 | US |
| Encorbio | https://encorbio.com/ | 1 | US |
| Good-biotech | http://www.good-biotech.com/ | 1,3 | Taiwan |
| Abnova | https://www.abnova.com/ | 1,3 | Taiwan |
| Sincerebio | http://www.sincerebio.com/ | 2,3 | Taiwan |
| Nabas | http://www.nabas.no/ | 1,2 | Norway |
| Gentian USA, Inc. | https://www.gentian.com/ | 2,3 | Norway |
| Medical biological laboratories | https://ruo.mbl.co.jp/ | 1,2,3 | Japan |
| Vonda Biotech | https://www.ovodanbiotech.com/ | 1,2,3 | Denmark |
| Bio-Connect* | https://www.bio-connect.nl/ | 1,2,3 | Netherlands |
| Hycult Biotech Inc. | https://www.hycultbiotech.com/ | 2,3 | Netherlands |
| Sanbio* | https://www.sanbio.nl/ | 1 | Netherlands |
| Abcepta | http://www.abcepta.com/ | 1,2 | UK |
| Biorbyt | https://www.biorbyt.com/ | 1,2,3 | UK |
| Agrisera | https://www.agrisera.com/ | 1,2,3 | Sweden |
| Immunsystem | http://www.immunsystem.com/ | 1,2 | Sweden |
| Dianova | https://www.dianova.com/ | 1,2,3 | Germany |
| Synaptic systems | https://www.sysy.com/ | 1,3 | Germany |
| Biozol* | https://www.biozol.de/ | 2,3 | Germany |
| Immune *Biosolutions* Inc. | https://immunebiosolutions.com | 1,2,3 | Canada |
| StressMarq Biosciences Inc. | https://www.stressmarq.com/ | 1,3 | Canada |

1: pAb; primary antibody; 2: sAb; secondary antibody; 3: Other products; (monoclonal antibody; tag antibody; and diagnostic kit); ***** Just as a seller and not a manufacturer

**Supplementary Table S6. Number of 10 top country for diagnostic and biotherapeutics IgY companies in the “Market” worldwide**

| Diagnostic | |  | | | Bio-therapeutics | |  | |
| --- | --- | --- | --- | --- | --- | --- | --- | --- |
| Country/  Region | Compan-ies (%) ^†^ | Products # (%) | | | Countries | Compan-ies (%) ^†^ | Products # (%) | |
|  |  | pAb | sAb | Other |  |  | Human* | Veterinary* |
| U.S | 27 (60) | 3008 (86) | 397(11) | 85 (3) | U.S | 4 (21) | 9 (15) | 4 (12) |
| Netherlands | 3 (7) | 10 (3) | 220 (72) | 76 (25) | South Korea | 3 (16) | 11 (18) | 25 (73) |
| Germany | 3 (7) | 146 (57) | 104 (41) | 5 (2) | Japan | 3 (16) | 19 (31) | 2 (6) |
| Taiwan | 3 (7) | 188 (75) | 2 (1) | 59 (24) | Romania | 2 (11) | 10 (16) | 1 (3) |
| Sweden | 2 (4) | 158 (68) | 71 (31) | 2 (1) | Canada | 2 (11) | 2 (3) | 0 (0) |
| Canada | 2 (4) | 132 (97) | 1 (1) | 3 (2) | Taiwan | 1 (5) | 7 (12) | 0 (0) |
| U.K | 2 (4) | 64 (58) | 42 (38) | 5 (4) | Argentina | 1 (5) | 0 (0) | 1 (0) |
| Norway | 2 (4) | 15 (58) | 3 (11) | 8 (31) | China | 1 (5) | 0 (0) | 1 (3) |
| Japan | 1 (2) | 2 (16) | 2 (16) | 8 (68) | Sweden | 1 (5) | 2 (3) | 0 (0) |
| Denmark | 1 (2) | 6 (50) | 4 (33) | 2 (17) | Denmark | 1 (5) | 1 (2) | 0 (0) |
| Total | 46 | 3729 (77) | 846 (18) | 253 (5) | Total | 19 | 52 (63) | 30 (37) |

pAb: Primary antibody; sAb: secondary antibody; Other products: (Monocolonal antibody; Tag antibody; and diagnostic kit); †: Top company in each country; #: Number of products (Polycolonal IgY); *: Human and Veterinary medicine

**Supplementary Table S7. Clinical trials of IgY in human medicine worldwide**

| Study Title | Conditions | Status | Interventions | Country/  Region |
| --- | --- | --- | --- | --- |
| IgY Efficacy on Periodontitis Patients | Chronic Periodontitis | Completed  (NCT02705885; not-applicable) | Dietary Supplement: lozenges containing IgY Dietary Supplement: Placebo IgY | Japan |
| Efficacy Study of IgY (Antibody Against Pseudomonas) in Cystic Fibrosis Patients | Cystic Fibrosis | Completed  (NCT01455675; phase 3) | Drug: IgY  Drug: Placebo | Austria |
| The Influence of IgY Max on Inflammatory Markers and the Gut Microbiome | Healthy | Completed  (NCT02972463; phase 1, phase 2) | Dietary Supplement: IgY, Other: Placebo | Canada |
| Safety, Tolerability, and pharmacokinetics of Anti-Severe Acute Respiratory Syndrome Coronavirus 2 (SARS-CoV-2) Chicken Egg Antibody (IgY) (COVID-19) | Covid-19 | Completed  (NCT04567810; phase 1) | Drug: anti-SARS-CoV-2 IgY Drug: Placebo | Australia |
| Anti-pseudomonas IgY to Prevent Infections in Cystic Fibrosis | Cystic Fibrosis | Completed  (NCT00633191; phase 1, phase 2) | Drug: Anti-pseudomonas IgY gargle | Sweden |
| Effect of Chicken Egg Antibody (IgY) on Patients With Chronic Gastritis | Helicobacter Pylori | Completed  (NCT02721355; not-applicable) | Dietary Supplement: GastimunHP | Vietnam |
| Effectiveness of GastimunHp Plus in Supporting the Treatment of Peptic Ulcer Disease With Helicobacter Pylori Infection | Helicobacter Pylori | Terminated  (NCT04025983; not-applicable) | Dietary Supplement: GastimunHp Plus  Other: Placebo | Vietnam |
| Oral AGY for Celiac Disease | Celiac Disease | Completed  (NCT01765647; phase 1) | Other: AGY | Canada |
| Study of Changes in Total Cholesterol Levels as a Function of Consuming a Supplement Designed to Improve Cardiovascular Health | Cholesterol  Hyperlipidemia | Unknown (NCT01890889; not-applicable) | Dietary Supplement: Ad-Chol-Pre  Dietary Supplement: Half-dose Ad-Chol-Pre | United States |
| Efficacy and Safety of IGN-ES001 in Chronic Widespread Pain With or Without Fibromyalgia | Chronic Widespread Pain  Fibromyalgia | Completed  (NCT03058224; not-applicable) | Drug: IGN-ES001  Drug: Parol 500 mg Tablets (acetaminophen) | Turkey |
| Impact of the Nutritional Product PTM202 on Acute and Long-Term Recovery From Childhood Diarrheal Disease | Diarrhea | Completed  (NCT02385773; not-applicable) | Dietary Supplement: PTM202  Dietary Supplement: Enfamil Puramino | Guatemala |
| A Randomized, Double-Blind, Placebo Controlled, Crossover Trial to Evaluate Safety and Efficacy of AGY in Celiac Disease | Celiac Disease | Recruiting (NCT03707730; phase 2) | Dietary Supplement: AGY, Other: placebo | Canada |
| Three Measures for the Prevention of Carious Lesions in Preschool Children | Dental Caries | Unknown (NCT02341352; phase 3) | Drug: fluoride varnish  Biological: Immunoglobulin Yolk Dietary Supplement: Probiotics | China |
| Treatment of Mild-moderate Clostridium Difficile Infection (CDI) (IM-01) | Clostridium Difficile | Recruiting (NCT04121169; phase 2 ) | Drug: IM-01  Chicken egg-derived anti-C.difficilepolyclonal antibodies | Canada |
| Sym021 Monotherapy, in Combination With Sym022 or Sym023, and in Combination With Both Sym022 and Sym023 in Patients With Advanced Solid Tumor Malignancies or Lymphomas | Metastatic Cancer Solid Tumor Lymphoma | Completed (NCT03311412; Phase 1) | Drug: Sym021; Anti-PD-1. (It is the first chicken derived mouse-cross-reactive antibody to enter clinical development)  Drug: Sym022  Drug: Sym023 | Denmark, Canada, United States |

**Supplementary Table S8. IgY products for the Chinese market***

| IgY Targets/Pathogens/ Diseases | Application/ Description | Species | No. of products |
| --- | --- | --- | --- |
| Human papilloma virus (HPV) | Anti-HPV | Human | 3 |
| *Helicobacter pylori* (HP) | Anti-HP | Human | 4 |
| SARS-CoV-2 virus | Toothpaste | Human | 2 |
| SARS-CoV-2 virus | Spray/ Liquid Dressing/ Hand Soap | Human | 4 |
| Gut microbes | Regulation of the intestinal microenvironment | Cat | 2 |
| Gut microbes | Regulation of the intestinal microenvironment | Dog | 1 |
| Gut microbes | Regulation of the intestinal microenvironment | Pig | 2 |
| Gut microbes | Regulation of the intestinal microenvironment | Cow | 1 |
| Avian Influenza Virus (AIV) | Anti-AIV | Chicken | 1 |
| Duck hepatitis virus (DHV) | Anti-DHV | Duck | 11 |
| Gosling Plague | Anti- Gosling Plague | Geese | 10 |
| Goose Parvovirus | Anti- Goose Parvovirus | Geese and ducks | 14 |
| Infectious bursal disease | Anti- infectious bursal disease virus | Poultry | 15 |

* Due to the data ownership, the names of the products and companies have not been provided

**Supplementary Table S9. Approved (vet) IgY drug and ongoing clinical trials in China**

| Clinical trial registration No./ New veterinary drug registration certificate No./ Product approval No. | Pathogen/Product name | Institution/Company | Status |
| --- | --- | --- | --- |
| ChiCTR2100045909 (2021) | Human papilloma virus | The Second Affiliated Hospital of Zhengzhou University | In progress |
| 2020039 | Refined Egg Yolk Antibodies to Lesser Goose Fever Virus | Liaoning Yikang Biological Co. | In progress |
| 2021030 | Refined yolk antibody to duck eutherian virus | Chongqing Sanjie Zhongxin Biological Engineering Co., etc. | In progress |
| 2021014 | Bivalent Yolk Antibody against Duck Viral Hepatitis (Type Ⅰ+Type Ⅲ) | Tianjin Rui Pu Biotechnology Co., etc. | In progress |
| (2011) New Veterinary Drug Certificate No. 43 | Freeze-dried yolk antibodies against infectious bursal disease in chickens | Liaoning Yikang Biological Co. | Completed |
| (2009) New Veterinary Drug Certificate No. 13 | Freeze-dried yolk antibodies against infectious bursal disease in chickens | Beijing Haidian Zhonghai Animal Health Technology Company | Completed |
| (2021) New Veterinary Drug Certificate No. 37 | Bivalent Yolk Antibody against Duck Viral Hepatitis (Type Ⅰ+Type Ⅲ) | Qingdao Yibang Biological Engineering Co. | Completed |
| (2016) New Veterinary Drug Certificate No. 7 | Duck Hepatitis I virus yolk antibody | Rui Pu (Baoding) Biopharmaceutical Co. and Tianjin Rui Pu Biotechnology Co. | Completed |
| (2016) New Veterinary Drug Certificate No. 50 | Duck Hepatitis A Virus Bivalent Egg Yolk Antibody (Type I+Type Ⅲ) | Yantai Green Leaf Animal Health Products Co., etc. | Completed |
| (2014) New Veterinary Drug Certificate No. 30 | Refined Egg Yolk Antibodies to Duck Hepatitis I Virus | Chongqing Yongjian Biotechnology Co Ltd and Tianjin Zhongsheng Challenge Bioengineering Co Ltd | Completed |
| (2012) New Veterinary Drug Certificate No. 30 | Freeze-dried egg yolk antibodies to duck viral hepatitis | Qingdao Baotek Biopharmaceutical Co., etc. | Completed |
| (2009) New Veterinary Drug Certificate No. 31 | Refined Egg Yolk Antibodies to Duck Viral Hepatitis | Luoyang Pleco Bioengineering Co. | Completed |
| (2016) New Veterinary Drug Certificate No. 68 | Goose Parvovirus Antibodies | Harbin Pharmaceutical Group Biovaccines Co., etc. | Completed |
| (2016) New Veterinary Drug Certificate No. 30 | Goose Parvovirus Antibodies | Rui Pu (Baoding) Biopharmaceutical Co. and Tianjin Rui Pu Biotechnology Co. | Completed |
| (2016) New Veterinary Drug Certificate No. 28 | Refined Egg Yolk Antibodies to Lesser Goose Fever Virus | Luoyang Pleco Bioengineering Co. | Completed |
| (2014) New Veterinary Drug Certificate No. 50 | Freeze-dried yolk antibodies against Goose Fever | Liaoning Yikang Biological Co., etc. | Completed |
| Veterinary Drug Sheng Zi 060132187 | Freeze-dried yolk antibodies against infectious bursal disease in chickens | Liaoning Yikang Biological Co. | Completed |
| Veterinary Drug Sheng Zi 160022064 | Antibodies Extracted From Chicken Egg-Yolk against Infectious Bursal Diseases | Luoyang Pleco Bioengineering Co. | Completed |
| Veterinary Drug Sheng Zi 150132064 | Antibodies Extracted From Chicken Egg-Yolk against Infectious Bursal Diseases | Qingdao Yibang Biological Engineering Co. | Completed |
| Veterinary Drug Sheng Zi 150432064 | Antibodies Extracted From Chicken Egg-Yolk against Infectious Bursal Diseases | Shandong Xinde Technology Co. | Completed |
| Veterinary Drug Sheng Zi 150132352 | Bivalent Yolk Antibody against Duck Viral Hepatitis (Type Ⅰ+Type Ⅲ) | Qingdao Yibang Biological Engineering Co. | Completed |
| Veterinary Drug Sheng Zi 020302264 | Duck Hepatitis I virus yolk antibody | Tianjin Rui Pu Biotechnology Co. | Completed |
| Veterinary Drug Sheng Zi 151822199 | Duck Viral Hepatitis Freeze-drying Antibodies | Shandong Lundu Biotechnology Co. | Completed |
| Veterinary Drug Sheng Zi 101042286 | Goose Parvovirus Antibodies | Sinopharm Yangzhou Weike Bioengineering Co. | Completed |
| Veterinary drug Sheng Zi 230102234 | Freeze-dried yolk antibodies against Goose Fever | Chongqing Yongjian Biotechnology Co. | Completed |
| Veterinary Drug Sheng Zi 151262286 | Goose Parvovirus Antibodies | Yantai Estin Animal Health Products Co. | Completed |
| Veterinary Drug Sheng Zi 150252286 | Goose Parvovirus Antibodies | Qilu Animal Health Products Co. | Completed |
| Veterinary Drug Sheng Zi 151182286 | Goose Parvovirus Antibodies | Qingdao Azure Biological Products Co. | Completed |
| Veterinary Drug Sheng Zi 080542286 | Goose Parvovirus Antibodies | Heilongjiang Zhengkang Biotechnology Co. | Completed |
| Veterinary Drug Sheng Zi 152982286 | Goose Parvovirus Antibodies | Weifang Huaying Biotechnology Co. | Completed |
| Veterinary Drug Sheng Zi 153882286 | Goose Parvovirus Antibodies | Shandong Delinor Bioengineering Co. | Completed |
| Veterinary Drug Sheng Zi 163582286 | Goose Parvovirus Antibodies | Shangqiu Meilan Biological Engineering Co. | Completed |
| Veterinary Drug Sheng Zi 060132234 | Freeze-dried yolk antibodies against Goose Fever | Liaoning Yikang Bio Co. | Completed |
| Veterinary Drug Sheng Zi 080072286 | Goose Parvovirus Antibodies | Harbin Pharmaceutical Group Biovaccines Co. | Completed |
| Veterinary Drug Sheng Zi 191132234 | Freeze-dried yolk antibodies against Goose Fever | Guangzhou Gretel Biotechnology Co. | Completed |
| Veterinary Drug Sheng Zi 020112286 | Goose Parvovirus Antibodies | Tianjin Zhongsheng Challenge Biotechnology Co. | Completed |
| Veterinary Drug Sheng Zi 151822286 | Goose Parvovirus Antibodies | Shandong Lundu Biotechnology Co. | Completed |
| Veterinary Drug Sheng Zi 020302270 | Goose Parvovirus Antibodies | Tianjin Rui Pu Biotechnology Co. | Completed |
| Veterinary Drug Sheng Zi 163062234 | Freeze-dried yolk antibodies against Goose Fever | Henan Houyi Biological Engineering Co. | Completed |
